# Structural and Chemical Hierarchy in Hydroxyapatite Coatings

**DOI:** 10.3390/ma13194447

**Published:** 2020-10-07

**Authors:** Karlis A. Gross, Christiane Petzold, Liene Pluduma-LaFarge, Maris Kumermanis, Håvard J. Haugen

**Affiliations:** 1 Biomaterials Research Laboratory, Riga Technical University, Faculty of Materials Science and Applied Chemistry, LV-1048 Riga, Latvia; kgross@rtu.lv (K.A.G.); liene.pluduma@gmail.com (L.P.-L.); mariskum@inbox.lv (M.K.); 2 Leibniz-Institute for New Materials, 66123 Saarbrücken, Germany; Christiane.Petzold@leibniz-inm.de; 3 Department of Biomaterials, Institute for Clinical Dentistry, Faculty of Dentistry, University of Oslo, 0455 Oslo, Norway

**Keywords:** electrical surface potential, hydroxyapatite coating, design, implants, self-assembly, hierarchy, biomaterials

## Abstract

Hydroxyapatite coatings need similarly shaped splats as building blocks and then a homogeneous microstructure to unravel the structural and chemical hierarchy for more refined improvements to implant surfaces. Coatings were thermally sprayed with differently sized powders (20–40, 40–63 and 63–80 µm) to produce flattened homogeneous splats. The surface was characterized for splat shape by profilometry and Atomic force microscopy (AFM), crystal size by AFM, crystal orientation by X-ray diffraction (XRD) and structural variations by XRD. Chemical composition was assessed by phase analysis, but variations in chemistry were detected by XRD and Raman spectroscopy. The resulting surface electrical potential was measured by Kelvin probe AFM. Five levels of structural hierarchy were suggested: the coating, the splat, oriented crystals, alternate layers of oxyapatite and hydroxyapatite (HAp) and the suggested anion orientation. Chemical hierarchy was present over a lower range of order for smaller splats. Coatings made from smaller splats exhibited a greater electrical potential, inferred to arise from oxyapatite, and supplemented by ordered OH^−^ ions in a rehydroxylated surface layer. A model has been proposed to show the influence of structural hierarchy on the electrical surface potential. Structural hierarchy is proposed as a means to further refine the properties of implant surfaces.

## 1. Introduction

When an implant is placed into the human body, the surface determines the interaction with the biological system and further integration. Thus, hydroxyapatite coatings on bone implants play a critical role for the integration with bone, and so consideration needs to be directed to the surface characteristics. Surface features include topography [1,2,3,4,5], chemistry [6,7,8] and charge [9,10]; these measures must be quantified at the micro-and nano-level [11,12,13]. Detailed surface characterization will show how thermal spraying can change the topography and surface electrical potential of hydroxyapatite coatings, and how the design of joint replacement surfaces could include a consideration of chemical and structural hierarchy [14].

**Figure 1:**
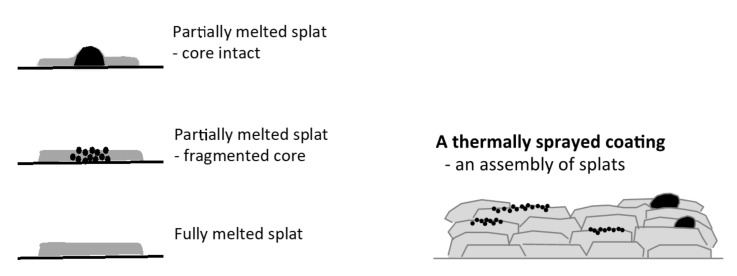
The variety of splats displaying the building block and their assembly in a thermal spray coating: a splat with a raised center from a partially melted particle, a micro-rough splat from a partially melted particle and a smooth splat from a melted particle. Black represents the unmelted particle fraction.

The surface topography/texture of a hydroxyapatite made by thermal spraying can be explained by focusing on the building blocks of the coatings-individual splats. An incompletely melted particle can produce a raised center if the core remains intact or a micro-rough splat if the particle core fractures upon impact, Figure 1. Both types of splats will introduce variations to the splat topography and microstructure. A homogeneous splat is needed from a completely melted particle to provide the best starting point for producing a homogeneous coating to allow optimization of the surface.

A homogeneous splat—the reproducible building block for coatings—will allow further design of the coating. Although the standard for orthopedic coatings requires the crystallinity to be above a threshold [15], hydroxyapatite can be made amorphous [16] or completely crystalline [17]. The uniformity is key for reproducibility of properties and performance as well as further optimization of the microstructure. The goal is to create a more uniform coating from the complete melting of particles, and apply Kelvin probe atomic force microscopy to probe for differences stemming from the change in microstructure.

In this work, we will characterize the structure (topography, splat, crystal size) and chemistry of thermally sprayed hydroxyapatite coatings made from powders of three different particle size distributions, to explore the change in topography and electrical potential. We hypothesize that smaller powder particles will result in a more homogeneous coating due to more uniform particle heating in the thermal spray plume. Structural hierarchy will be illustrated within crystals, splats and the coating. We suspect a different hierarchy for smaller particles than for larger particles. The objective is to show the formation of a homogeneous building block, and reveal a structural hierarchy that could be used for improvements in hydroxyapatite-coated implants.

## 2. Materials and Methods

Analysis of the coating surface and not single splats on metal is necessary to report on the surface characteristics of an implant surface. Earlier studies focused on single splats collected on substrates from a “wipe test” [18] or from passing through a fine aperture [19,20]. Splats solidified on metallic substrates do not represent the shape, nor the microstructure of splats solidified on previously deposited hydroxyapatite splats. Melted hydroxyapatite powder particles offer the potential to spread out better on previously deposited splats due to (a) an increased wettability, and (b) a lower thermal conductivity of previously deposited hydroxyapatite [21]. Therefore, the investigation will be on a coating instead of single splats, that have a different microstructure.

### 2.1. Coating Preparation

Coatings were flame-sprayed with spray-dried HAp powder (CAM Bioceramics, Leiden, The Netherlands). Powder was classified by wet sieving to three different particle size distributions (20–40 µm, 40–63 µm and 63–80 µm), dried and then re-sieved to restore powder flowability. The powder was transported in a dry-air carrier gas to a flame in the Metco 6P flame spray torch (Sulzer-Metco, Winterthur, Switzerland) fuelled by oxygen and acetylene gas. Coatings were made on grit-blasted pure titanium substrates.

Based on conditions for producing melted particles, a spray distance of 10, 12 and 15 cm was chosen for the 20–40 µm, 40–63 µm and 63–80 µm powder, respectively. The larger particles required a longer residence time for melting the larger particle volume. A preheat of 400 °C was chosen since it exceeds the 100 °C temperature for making rounded splats [17] and also attains the 400 °C required to prevent moisture adsorption [22] during the coating process.

### 2.2. Measurement of Purity, Phase Composition and Surface Electrical Potential

Phase and chemistry were measured over two length scales. X-ray diffraction provided a global assessment of coating surface, but micro-Raman spectroscopy probed the bonding at the micron-sized level. Trace elements in the hydroxyapatite were detected using a SCIEX ELAN DRC-inductively coupled plasma–mass spectrometer (ICP-MS) (PerkinElmer, Waltham, MA, USA). Argon with a purity of 99.999% was used as a carrier gas (AGA, Riga, Latvia). The powder with a weight of 0.3 g was digested in an oxidizing acidic medium consisting of 65% nitric acid (Suprapur, Merck, Darmstad, Germany) and 30% hydrogen peroxide (Suprapur, Merck, Darmstad, Germany) and then homogenized in an ultrasonic bath (Sonorex RK100 BANDELIN electronic GmbH and Co. KG, Berlin, Germany). Mineralization was carried out in a closed vessel microwave digestion system (Anton Paar 300, Anton Paar GmbH, Graz, Austria). A total of 5 measurements were taken and the average value calculated.

X-ray powder diffraction (XRD) checked for preferred crystal orientation and confirmed phase purity. For crystal orientation, characterization was conducted on the coating to see if a change occurred in the relative peak intensities. To determine the phase purity, a random crystal orientation was preferred and so the coating was triturated to a fine powder in a mortar and pestle before conducting XRD. X-ray diffraction patterns were obtained by a D8 ADVANCE diffractometer (Bruker Corporation, Billerica, MA, USA) recorded from 5° to 60° using Cu Kα radiation (λ = 1.54180 Å generated at 40 mA and 40 kV) at a 0.02° step size.

Raman spectra were obtained by the InVia micro-Raman spectrometer (Renishaw, Wotton-under-Edge, Gloucestershire, United Kingdom) with a He-Ne (633 nm) red laser excitation through a 1800 mm^−1^ grating in a backscattering geometry. The laser was operated at 10 mW. The spectrometer was calibrated with silicon, giving a Raman line at 520 cm^−1^. Spectra were acquired for 40 s from the center of the splat, and at opposite sides on the outer edge of the splat. Spectra were normalized by multiplying all intensity values with a factor to reduce the left peak at 446 cm^−1^ to unity. This would allow any variations in the OH^−^ content at 963 cm^−1^ to be readily detected.

An atomic force microscope (Solver-Pro NT-MDT, Moscow, Russia) with a Kelvin probe force microscopy function measured the surface electrical potential. Five measurements were obtained for each sample and the average value was calculated. The size of each scan was 2 × 2 µm. Each image consisted of 256 × 256 data points. A semicontact method was used. The average electrical potential value was calculated for each sample from the average of each scan.

### 2.3. Measurement of Micro- and Nano-Topography

Topography was assessed over two length scales with stylus/probe measurement methods. Profilometry, as the conventional surface engineering method, determined the splat shape at a larger length scale, while atomic force microscopy measured more detail of the splat edge and splat surface.

Profilometry was performed (Talysurf Intra 50 contact profilometer, Taylor-Hobson, Leicester, England) with a spherical stylus of 2 μm diameter and an included angle of 90°. A map with an area of 200 × 200 µm² (400 × 400 data points) was produced by TalyMap Expert software (Taylor Hobson, Elancourt, France) as a 3D view or a photosimulation. The topography was plotted in a 2D view with a corresponding line profile.

The height of selected splats was determined from line profiles made through the center of the splat by measuring the vertical distance between the reference baseline outside of the splat and the highest point on the splat. Line profiles were made at 45° incremental rotations and an average calculated from profiles collected at 8 different rotations. This was easily conducted for the 20–40 µm source powder, but the large splat area resulting from the larger particle sizes made it difficult to determine a baseline, and so the splat height for the largest particle sizes was not calculated.

**Figure 2:**
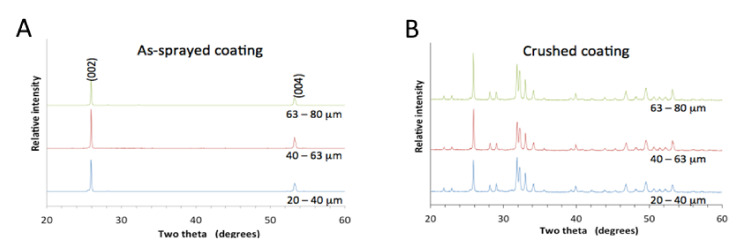
X-ray diffraction of (**A**) coatings showing a <001> crystal orientation and of (**B**) crushed coatings displaying peaks characteristic of hydroxyapatite. Coatings were made from 20–40 µm powder, 40–60 µm powder and 60–80 µm powder.

Topography of thermal spray HAp coatings was analyzed with an atomic force microscope (MFP 3D; Asylum Research, Santa Barbara, CA, USA) in air with fast scan direction perpendicular to the cantilever (probes: OMCL-AC160TS-W2, Olympus Optical Co. Ltd., Tokyo, Japan) to avoid buckling. Height of the individual splats and HAp crystal size on the splat surfaces were analyzed. Splat heights were determined from profiles of scans of 50 µm × 50 μm (N = 23) from splats of the 3 groups. Height differences were measured from the smoothened profile data (N = 119 edges). For crystal size analysis, entire splats of the group 20–40 µm were imaged at 50 μm × 50 µm (N = 5). Subsequently, the AFM tip was positioned in the center (C), edge (E) or transition zone (T) of the splat. The positions were assigned for each splat individually based on the previous scan at 50 μm × 50 µm, Figure 2. On each position, two images were scanned: 1 × 1 µm^2^ and 5 × 5 µm^2^. The image centers were defined as follows: C—center of the splat, E—3 µm from the border of the splat, T = (C−E)/2. The distance between the centers of the scans at the respective positions was between 8 µm and 10 µm: splat sizes were chosen such that the scan areas of C, T and E would not overlap. Crystal sizes were measured with Gwyddion (Version 2.3, http://gwyddion.net) from the image sized 1 × 1 µm^2^ or 5 × 5 µm^2^, depending on the crystal size and correlated to their respective positions.

### 2.4. Statistics

All data were analyzed with Origin 8.5 (Origin Lab Inc., Northampton, MA, USA). The significance was determined with One Way ANOVA in case of normally distributed data, or Kruskal–Wallis One Way Analysis of Variance on Ranks in case of nonparametric data. The results are given as median ± interquartile range (IQR). Statistical significance is indicated as *P* < 0.05.

## 3. Results

### 3.1. Purity and Phase Analysis

All coatings made from the differently sized particles exhibited oriented hydroxyapatite crystals within splats. For a random crystal orientation, the most intense peak in the XRD pattern was expected at 31.8°, but the highest peak at 26.1° indicated oriented crystals, as the diffraction condition was only satisfied for crystals with a <00*l*> alignment, Figure 2A. The next most intense XRD peak located at 53.1° originated from the same crystal orientation. The most intense peak stemmed from the (002) crystal planes, indicating diffraction from half the height of the unit cell, but the second peak associated with the (004) crystal plane originated from diffraction within a quarter of the unit cell height. Given the definition of crystallinity for thermal spray coatings based on the amount of crystalline material where amorphous and crystalline phase may be present [16], this diffraction pattern represented a 100% crystalline coating, with crystals oriented such that the <00*l*> direction was perpendicular to the surface.

The crushed and triturated coatings showed peaks characteristic of an apatite phase, indicating that only apatite is present in the textured coating. The (002) peak appeared at a slightly greater intensity than expected for a complete random crystal orientation but this did not deter us from seeing that only apatite was present (the (002) peak should be at 25% intensity compared to the most intense (211) peak at 31.8°), Figure 2B.

Purity of the coating was reported in terms of the trace elements and the Ca/P molar ratio. Hydroxyapatite contained few trace elements as shown by the ICP-MS results. The largest concentrations above 1 ppm included 68.1 ppm Sr, 5.2 ppm Ni, 4.0 ppm Mn, 1.3 ppm Ba and 1.2 ppm Zn. The Ca/P molar ratio based on the X-ray diffraction and the absence of impurity phases [23] was deemed as 1.67.

**Figure 3:**
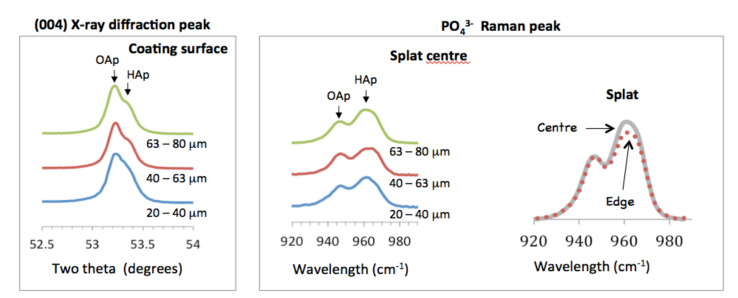
Oxyapatite and hydroxyapatite shown in X-ray diffraction patterns and Raman spectra. The oxyapatite peak was normalized in both the X-ray diffraction patterns and Raman spectra for ease of comparison. Raman spectra for the coatings were obtained from the middle of the splat. Raman spectra from the large splat made from 63–80 µm powder was taken in the center (^___^) and edge (^…^) of the splat.

A detailed examination of the Raman peak centered at 955 cm^−1^ indicated the presence of oxyapatite (OAp). This was supported by the doublet in the (004) XRD peak that was seen at about 53.3°, Figure 3. The left shoulder represented oxyapatite [24] and was more intense for the coating made from finer powder (compared to XRD of coatings made from 20–40 µm powder and 63–80 µm powder). The Raman spectra showed a peak at 963 cm^−1^, assigned to hydroxyapatite, and a peak at 946 cm^−1^, representing oxyapatite [25]. The hydroxyl peak was 31% more intense than the oxyapatite peak for the coatings made from the smaller powder fractions, but 44% more intense for the largest powder.

Raman spectroscopy showed that the hydroxyl concentration in splats was higher at the center than at the rim of the splats. For the coating made from the smallest powder (20–40 µm), the 963 cm^−1^ peak height was 35% more intense relative to the 946 cm^−1^ peak height. At the edge of the splat, the 963 cm^−1^ peak height was 31% more intense on opposite ends of the splat. This showed that the splat could have contained a lower OH^−^ concentration at the edge compared to the splat center, Figure 3. This effect was clearly shown for splats made from larger particles. For the coating made from 63–80 µm powder, the hydroxyapatite peak (at 963 cm^−1^) at the center was 52% relative to the oxyapatite peak (at 946 cm^-1^), but only 40% on opposite ends of the splat.

### 3.2. Electrical Surface Potential

**Figure 4:**
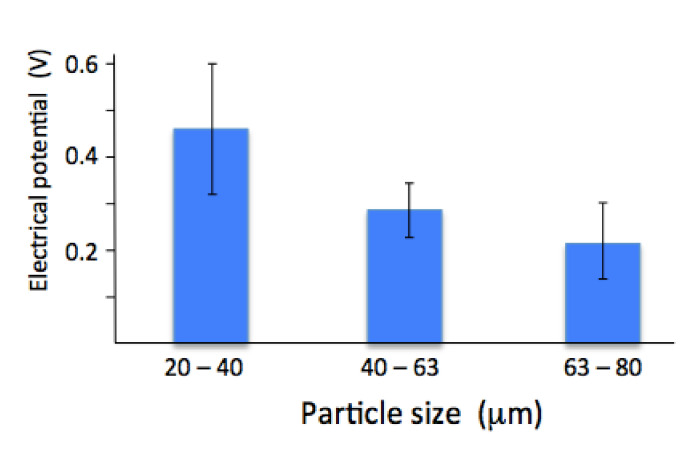
Electrical surface potential measured by Kelvin probe AFM on coatings made from 20–40 µm, 40–60 µm and 60–80 µm powder.

The surface electrical potential measured by Kelvin probe atomic force microscopy showed a change with particle size. Coatings made from a smaller particle size gave the largest surface electrical potential. There was a noticeable drop with coatings made from larger particles (both the 40–60 µm and 60–80 µm powders). The largest particle size gave the smallest surface potential, Figure 4. Since the crystal size was similar for all the coatings (see Table 1), the difference is not related to crystal size or grain boundary area.

### 3.3. Splat Topography—Height, Edge Geometry and Flatness

**Figure 5:**
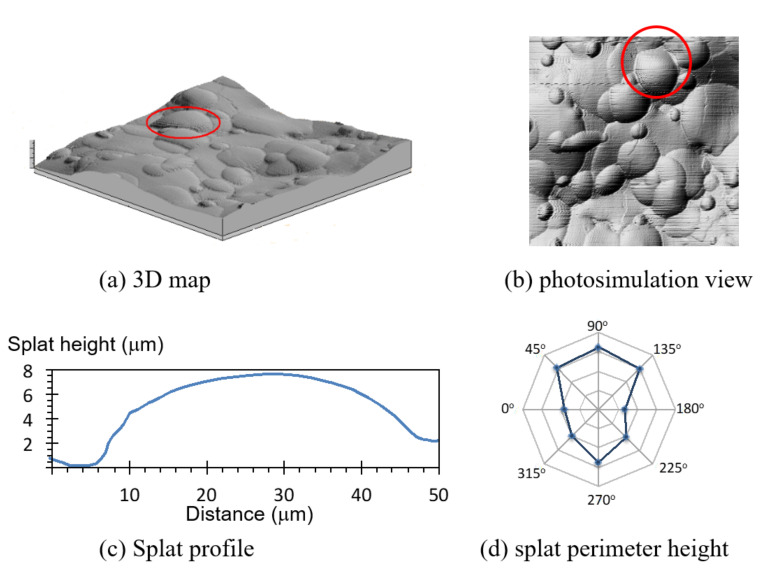
A topographical map from 20–40 µm droplets measured by profilometry (**a**) in a 3D map of a 200 µm × 200 µm section and (**b**) in a photosimulation view. A more detailed analysis of the circled Splat shows (**c**) a profile view of a Splat at a 0°–180° section, and (**d**) the Splat height at different rotations. The orientation of (**b**) and (**d**) are identical to show the influence of surrounding Splats on the measured Splat height. The webs on the radar plot start at 0, with height increments of 4 µm.

Splats populating the three different coatings were rounded, as opposed to splashed splats that have an erratic circumference, and more prevalent in plasma-sprayed coatings. The smaller splats were identified and measured with the most ease; larger splats overlapped more frequently and were more commonly covered with smaller splats. Most splats were circular, but some exhibited an elongated shape from the underlying terrain. The unevenness of the coating surface is best viewed in the 3D map (Figure 5a), but the splat shape is best surveyed in the photosimulated view of the coating, Figure 5b.

Splat height at the circumference varied in response to the underlying surface contour and surrounding splat proximity. A line profile on a close-to-horizontal reference plane showed a similar splat height at opposite ends (orientation 0°–180°; Figure 5c). Line profiles taken at other orientations —through the center of the splat (marked in Figure 5b)—displayed different splat heights at the opposite ends (Figure 5d). A shorter-perimeter splat was registered where there was interference from neighboring splats; as seen at 0°, 180°, 225° and 315° (Figure 5d). Conversely, taller splats were recorded when the reference line outside the splat was at a lower point. The highlighted splat (Figure 5a,b) formed on a ridge leading to an apparent higher splat boundary at 45°, 90°, 135° and 270°.

**Figure 6:**
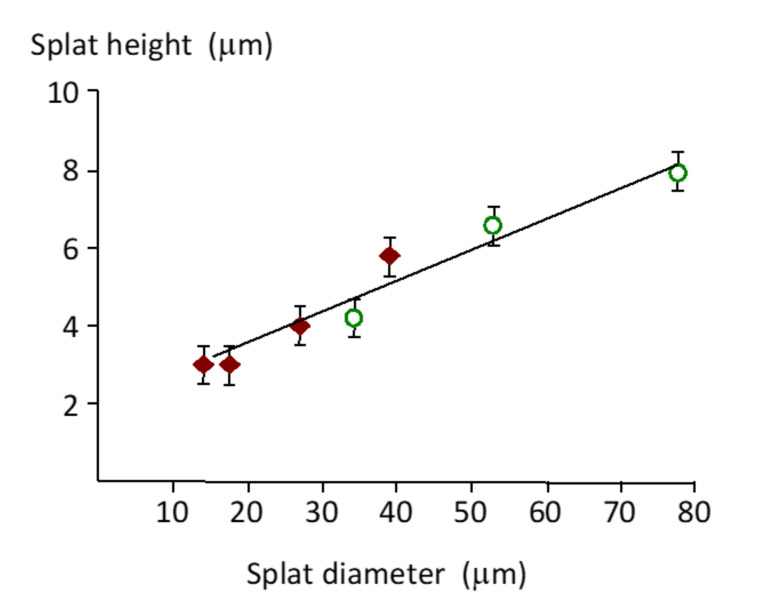
Variation of splat height (h) with splat diameter (d), according to the line of best fit, h = 0.083d + 1.743 (R^2^ = 0.98) in the splat size range of 15 µm to 80 µm. Data with diamond symbols were sourced from the coating made from 20–40 µm particles, and the data with open circles came from the coating made from 40–60 µm particles.

Splat height increased with a larger splat diameter (Figure 6). Small 15 µm diameter splats were 3 µm high, while a splat with a diameter of 80 µm (about 5-times larger) was 7.8 µm high (2.6-times higher). This trend was also observed for coatings made from 20–40 µm and 40–60 µm powders. Splat height was difficult to measure with large splats due to interference from the underlying irregularities.

**Figure 7:**
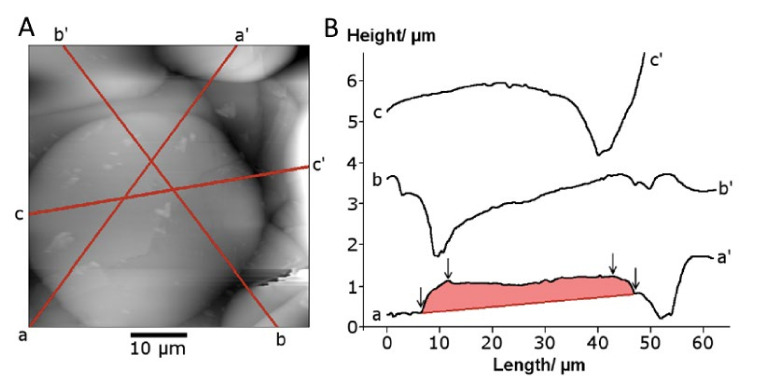
AFM scans of (**A**) a splat and (**B**) the traverse lines (a, b and c) to produce profiles of the splat shown as traces a–a’, b–b’ and c–c’ of a splat. The shaded trace a-a’ was used for splat height analysis.

The two stylus/probe measurement methods—profilometry and atomic force microscopy—recorded the splat geometry at different levels of sensitivity. The profilometer stylus with a large 90° included angle could not accurately determine the splat perimeter shape, producing a gradual decrease in height. The splat edge geometry was more accurately reflected with the 18° included angle (i.e., 9° half angle for scans at 90° angle relative to the cantilever orientation) of the AFM tip, Figure 7. The average splat height was determined to be 1 ± 0.6 μm for a 40 µm splat (see a–a’ traverse in Figure 7B). This splat appeared to be seated in an underlying cavity, with the line a–a’ representing the highest points.

Two types of splat profiles were observed for smaller splats: a flattened disk with a raised center and splats with a lowered center compared to the edges. A raised center, comparable to a flattened hemisphere, was more frequently observed for splats on a relatively flat background. The splat with a flat surface was formed on a slope (b’–b in Figure 7) that preferentially directs the center of mass towards the lowest point.

Splats larger than 50 µm displayed an undulating surface reflecting the underlying coating surface—a raised center could not be detected. If a raised center was formed, then this blended in with the many undulations on the splat surface.

### 3.4. Crystal Size

**Figure 8:**
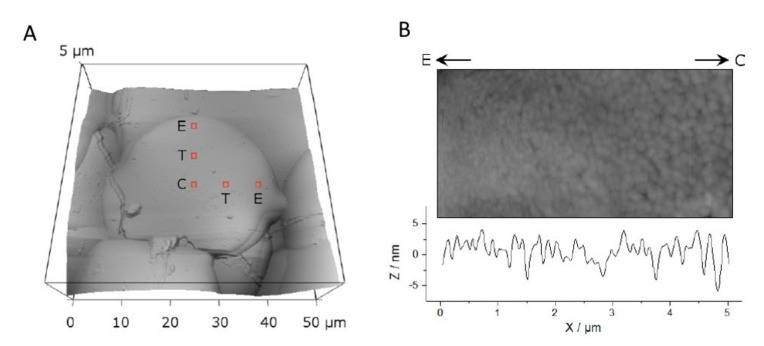
A splat visualized by AFM (**A**) outlining three analysis positions and (**B**) showing an enlarged view.

Variation in the crystal size occurred both in differently sized splats and also within the splat. Small splats showed an average crystal size of 200 µm (made from 20–40 µm particles), but larger splats exhibited an average of 260 µm-sized crystals (from 63–80 µm particles), Table 1. The crystal size was relatively uniform over the majority of the splat, Figure 8. Within a small splat the central region (position C) showed significantly larger crystals (225 µm) compared to the very edge (position E) where crystals with a diameter of 100 µm were observed. The central area of the splat is where crystal growth occurs first [26], explaining the larger crystals.

**Table 1:** Crystal size for splats made from differently sized particles, and within a splat at positions E, T and C (median ± interquartile range, *P* < 0.05 indicates differences between measurement groups outside the rejection region for a significance level of *P* = 0.05).

Particle Size (µm)	N	Crystal Size (nm)	IQR	*P* < 0.05
20–40	27	200	0.038	40–60, 60–80
Edge, E	10	100	0.012	C, T
Transition, T	11	215	0.020	E
Center, C	6	225	0.016	E
40–60	33	240	0.032	20–40
60–80	19	260	0.063	20–40

Along the direction from the center (C) to the edge (E) to reveal a gradual increase in crystal size towards the center.

## 4. Discussion

The combination of homogeneous splats and detailed analysis techniques allowed more detailed characterization. Structure (surface topography, splat topography and crystal structure), chemistry (crystal structure and bonding) and electrical potential will be discussed in turn and the information collated to reveal the structural and chemical hierarchy within coating surfaces. Splats will be referred to as hydroxyapatite splats since the oxyapatite quickly takes up water vapor to form a thin outer hydroxylated film, but reference will be made oxyapatite or hydroxyapatite when the splat is discussed in more detail.

### 4.1. Topography of Hydroxyapatite Splats

While previous work used roughness to describe the coating smoothness [27,28], this generic measure of surface undulations has not identified the individual splat and finer attributes of the splat. Initial work on thermally sprayed materials was directed at ensuring rounded splats, without splashing [29,30,31,32]; this work has shown that the true surface-splats on titanium have a rim at the edge [33]—splats deposited directly on other HAp splats are flat in comparison.

It is interesting to note a linear increase in the splat height with the splat diameter for the small splats investigated here. Larger particles had an unmelted core, and formed a splat with a higher center and so made it difficult to measure the splat height. Further reports should consider mapping the splat microstructure (crystal size, crystal orientation and chemistry) with the particle size.

The recorded splat shape depends on the scanning probe internal angle. A large included angle for the profilometer did not accurately measure the splat shape. Previous measurement with a Berkovich probe on a nanoindentor with a larger included angle showed a tapered edge [34], similar in shape to the splat edge recorded with the profilometer probe, Figure 6. In the 3D map or photosimulation made from the profilometer data, all splats appeared tapered. The image from an AFM probe with the small included angle showed a steeper edge, more representative of the true situation. The higher sensitivity of the AFM tip has also been reported for inkjet-printed, splat-shaped deposits [35]. The rounded splat edge shown by the AFM probe has also been found in cross-sections of focused-ion-beam-prepared splats [36].

### 4.2. Variations in Chemistry on the Coating Surface

Both oxyapatite and hydroxyapatite were identified from the different unit cell heights as shown by the X-ray diffraction doublet, and from the two types of phosphate bonding in the Raman peak. The XRD peak doublet implies that a hydroxyapatite-oxyapatite solid solution did not form, that could otherwise be observed as a single, wide peak. A single, wide peak is not expected due to OH^−^ removal from the droplet outer layer followed by rapid solidification as oxyapatite. Oxyapatite then takes up water vapor on the surface and adds a surface hydroxyapatite layer to supplement any hydroxyapatite that may already be present in the splat core.

There are two distinguishable scenarios that could lead to distinct oxyapatite and hydroxyapatite regions. In the first situation, the completely dehydroxylated droplet flattens, solidifies as oxypatite and then incorporates water from the atmosphere to form a hydroxyapatite shell. This agrees with the findings from earlier studies showing a greater OH^−^ concentration on the coating surface [24]. In the second situation, the core of the droplet retains hydroxyl ions, unable to release OH^−^ ions through the larger droplet diameter: the partially dehydroxylated droplet solidifies as oxyapatite and the outer layer converts to hydroxyapatite as water is included from the atmosphere. The first scenario leads to a hydroxyapatite skin surrounding an oxyapatite core, but the second scenario depicts a striated or banded structure to reflect the OH^−^ retained in the core and incorporated on the surface.

For the interpretation of the distribution of oxyapatite and hydroxyapatite it is important to consider that (1) the edge of the splat will always have the lowest OH^−^ content and (2) smaller splats will be more unlikely to contain OH^−^ ions in the splat.

A previous model based on X-ray diffraction patterns of the crushed coating, and not an analysis of the splat as conducted here, inconclusively suggested oxyhydroxyapatite at the core [37]. Our results do not support those findings. The results of this study clearly show two distinct spatial regions in the splat: an oxyapatite-rich region and a hydroxyapatite-rich region.

### 4.3. Surface Electrical Potential

The larger electrical surface potential from smaller splats could be explained by two factors. The first source of the field could come from oxyapatite [38]. Oxyapatite consists of alternative stacking of defects and O^2−^ ions in columns within the unit cell [39]. This alternating stacking arrangement with a negative charge at one height and a neutral charge at the other level, stacked together in the column of the unit cell, is similar to a dipole and provides an overall negative field. Since smaller splats contain more oxyapatite, one expects a higher surface electrical potential in smaller splats. The contributing factor to the surface electrical potential could arise from stacking of aligned OH^−^ ions in the outer layer. The ordering of the OH^−^ ions may occur spontaneously under the influence of the underlying negative field in oxyapatite, causing the hydrogen to enter the column first and be attracted by the underlying negative charge. Consequently, the supposedly oriented OH^−^ ion layer forms a dielectric plug in the outer surface, and delays continued OH^−^ inclusion deeper into oxyapatite over time. This change in OH^−^ content with depth-over-time needs more refined tools such as scanning near-field optical microscopy (SNOM) for the simultaneous measurement of the OH^−^ content and the surface electrical potential [40].

**Figure 9:**
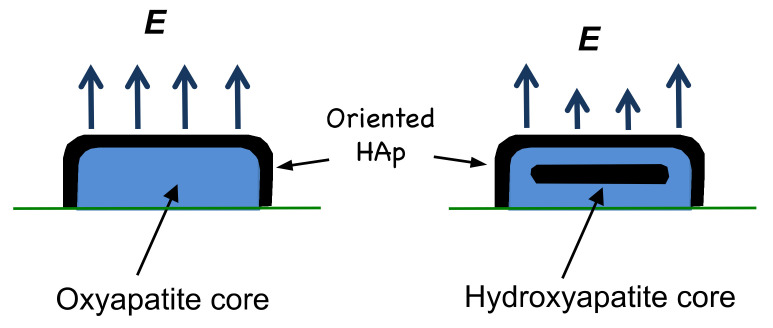
The hydroxyapatite and oxyapatite regions within a splat, where an oxyapatite core occurs for smaller splats and a hydroxyapatite core is found in larger splats. Both splats are normalized for ease of comparison. An 80-µm-sized droplet, depicted to the right with a HAp core, would be 4.3-times larger in diameter and a 3-times higher, if extrapolated from the data shown in Figure 8.

Given the observation of a larger surface electrical potential from smaller splats, it would appear that the origin of the potential in thermal-spray coatings is associated with oxyapatite, with a concomitant contribution from the outer hydroxyapatite layer. This is best illustrated in a comparison of a small splat and a large splat (normalized to emphasize the OH^−^-rich and the OH^−^-depleted layers) to show the chemically different areas and the resulting surface electrical potential. A stronger field is expected from an oxyapatite core and this is shown with larger arrows. Smaller arrows from the splat with a hydroxyapatite core are assumed to arise from the randomly oriented OH^−^ ions in the core, thus creating an insulator that dampens the electrical field effect from the underlying oxyapatite layer, Figure 9.

### 4.4. Structural and Chemical Hierarchy

The hierarchy, both structural and chemical, will impact the charge created within the coating, that has been shown to impact bone formation [41]. A knowledge of factors that influence the hierarchy will add an extra level of control for the design of coatings. A model is proposed for the hierarchy in the coatings.

**Figure 10:**
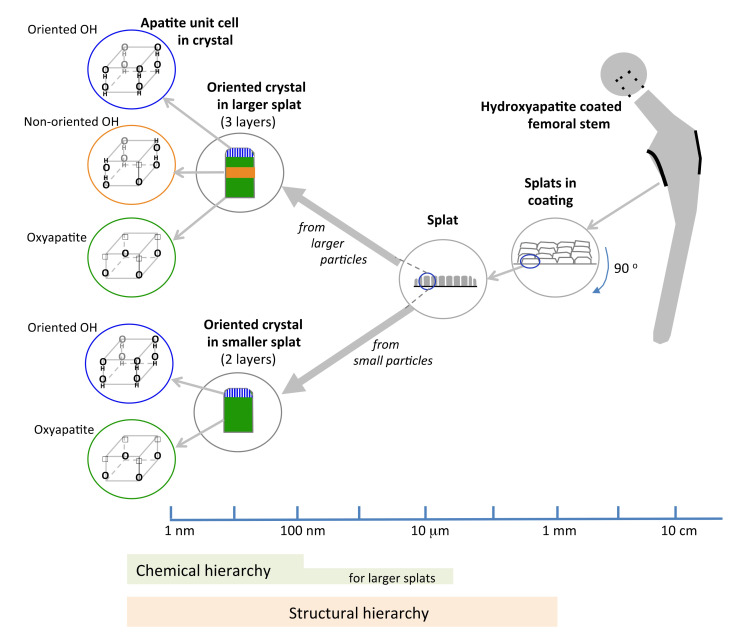
Proposed schema summarizing the five levels of structural hierarchy and three levels of chemical hierarchy in hydroxyapatite-coatings made from different sized splats.

Characterization of splat-designed coatings suggested hierarchy over five orders of magnitude from the angstrom level to the millimeter level [42]. A hierarchy was found both in structure and chemistry. This hierarchy stemmed from the control of particle processing (particle heating, splat size) and led to changes in crystal characteristics within the splat [42,43]. Structural hierarchy spanned from OH^−^ dipole arrangement in the unit cell at the angstrom level to the stacking of splats within the coating at the macrolevel (Figure 10), also seen by others [43]. Splats consisted of oriented crystals comprising of both oxyapatite and hydroxyapatite layers. The HAp layer on the splat surface arose from surface rehydroxylation [44]. The HAp lenticular layer in the core of the splat—only reported in larger splats—resulted from retained OH^−^ ions in the splat core (Figure 8 and Figure 9). The HAp in the core is expected to have a disorganized OH^−^ ion arrangement. Ordering of O^2^ and vacancies in the oxyapatite layer is surmised to cause ordering of OH^−^ ions in the upper/outer HAp layer, as indicated by the larger electrical surface potential, Figure 3.

The structural hierarchy includes OH^−^ orientation, the crystal structure, layers within the crystal, crystal assembly within the splat and splat assembly; the chemical hierarchy covers fewer successive levels of order. Smaller droplets are totally dehydroxylated before creating a splat and will display chemical order up to the crystal level. Larger droplets retain an OH^−^ core and will manifest a variation along the splat diameter, Figure 8, extending the chemical hierarchy up to the splat level, Figure 10, also reported by Tanaka et al. [45]. Above a critical particle size, the hierarchy increases.

This discussion assumes that phosphate is not preferentially volatilized from the outer droplet layer.

In the present study, the hierarchy is created by OH^−^ removal during particle heating, the assembly of aligned oxyapatite crystals in the microstructure during solidification and a self-assembly of OH^−^ ions from the adsorbed water in the air after deposition. To orchestrate these separate-but-interdependent events requires control over a series of thermodynamically and kinetically driven processes in a short time. The optimization of events over a time-scale and length-scale has provided the first step to hierarchically designed implant materials.

### 4.5. Design Opportunities for Hydroxyapatite Implants

Previous coating designs were only required to satisfy requirements of hydroxyapatite purity, hydroxyapatite crystallinity and coating adhesion [46]. Unmelted particles have contributed to particle release [47] followed by signs of inflammation [48,49,50,51]. A designed microstructure can avoid these potential problems and provide a functional biocompatible material.

A homogeneous coating with oriented crystals has shown better remodeling based on osteoclast resorption: a comparison between dentine, a hydroxyapatite coating with oriented crystals, and a sintered hydroxyapatite showed a similar osteoclast population on dentine and thermally sprayed hydroxyapatite [17]. The reason for resorption remains to be determined from the nanotopography, grain size, oriented crystals and surface electrical potential.

An implant surface charge made by structural and chemical hierarchy could be used to favor protein attachment [52], cell adhesion [53] and bone growth [10] and provide docking capabilities for biological species or drugs. Adjusting the surface charge of hydroxyapatite can support protein attachment. Previous attempts for attaching proteins onto hydroxyapatite have charged hydroxyapatite by initial attachment of selected amino acids [54]. Perhaps designing hydroxyapatite to include a surface charge may be a better and simpler option for achieving protein attachment, but this remains speculation at this moment.

## 5. Conclusions

Homogeneous splats have allowed more detailed characterization of coatings with attention to the splat shape, splat thickness, splat edge, splat crystal size, splat chemistry and electrical surface potential.

Different levels of hierarchy were found within the splat-assembled coatings at the structural and chemical level. Firstly, all splats exhibited relatively even thickness, disk-like structures with steep edges. Crystals within splats were oriented and appeared to be twice as large in the middle of the splat compared to the splat edge. A small increase in overall crystal size occurred for larger splats, but this was less marked than the change in crystal size with position in the splat. At the lowest level of structural hierarchy, layers of oxyapatite and hydroxyapatite were found from the change in unit cell dimensions by X-ray diffraction and chemical bonding by Raman spectroscopy.

Chemical hierarchy appears horizontally and vertically within the splat. An oxyapatite core in smaller splats indicated complete removal of hydroxyl ions during heating, but a hydroxyapatite core in larger splats only showed partial hydroxyl depletion. The greater surface electrical potential from smaller splats suggests that oxyapatite forms a dielectric that induces hydroxyl ion orientation on the splat surface.
